# Perceptions of Food Marketing and Media Use among Canadian Teenagers: A Cross-Sectional Survey

**DOI:** 10.3390/nu16172987

**Published:** 2024-09-04

**Authors:** Emily Truman, Charlene Elliott

**Affiliations:** Department of Communication, Media and Film, University of Calgary, Calgary, AB T2N 1N4, Canada; emily.truman@ucalgary.ca

**Keywords:** food marketing, digital media, teenager, youth, adolescent, persuasive power, food brand, media use, Canada, attitudes

## Abstract

Despite the prevalence of digital food marketing to teenagers and its potential impact on food preferences and consumption, little is known about the specific food advertisements teenagers see in Canada and how they perceive them. Further, few studies consult teenagers directly about their perceptions of teen-specific food marketing content. To shed light on such issues, this study examines perceptions of food marketing and self-reported media use of Canadian teenagers via an online survey. Four hundred and sixty-four teenagers (ages 13–17) participated. Overall, teenagers identified Instagram and TikTok as the most popular social media platforms. The top food or beverage brands that teens felt specifically targeted them were McDonald’s, Starbucks, Coca-Cola and Tim Hortons, while Instagram was deemed the most important media platform when it comes to teen-targeted food marketing. Teens deemed “celebrity” and “visual style” as the most important (food and beverage) advertising techniques when it comes to persuading teenagers to buy. Overall, the study provides insights into teen media use and brand preference, including the brands teens feel target them most directly and what they consider to be salient in terms of the food advertising they see. It also provides valuable details for researchers seeking to further identify and measure elements of teen-targeted food marketing.

## 1. Introduction

Despite the prevalence of digital food marketing to teenagers, little is known about what food messaging Canadian teens see and how they perceive it. Food marketing to teenagers is a growing area of interest given the targeted advertising for unhealthy products directed at this group, and the potential impact of these ads on food attitudes and behaviours [1,2,3]. Indeed, recent studies suggest that food marketing to teenagers impacts outcomes such as brand awareness, food preferences and purchase choices, as well as consumption [4,5,6]. While the potential effects on teens are unique given their developmental stage [7,8], few studies consult teenagers directly to explore their perceptions of teen-specific food marketing content. Even more rare is research that considers teen perceptions of the relative importance of various media platforms used to reach them with food marketing messages [9,10,11]. A better understanding of teen perceptions of targeted food messaging is needed in order to explore its potential impacts.

When it comes to teenagers in Canada, limited research measures teen opinions about the food marketing messages that reach them. Currently, only two cross-sectional survey studies examine food messaging to Canadian teens, focusing on exposure [12] and a range of impacts on food attitudes and behaviours [13]. While these studies use secondary self-reported teen data, only one study inquires *what* teens saw (i.e., ad content) and *where* they saw it (i.e., media platforms) [12].

To shed more light on teen-targeted food messaging, this study examines perceptions of food marketing and self-reported media use of teenagers (ages 13–17) in Canada. The research fills a gap when it comes to outcomes measured in current survey literature, with a focus on the “power” food marketing content (i.e., specific techniques used within marketing messages that work to persuade) and media platforms. As such, it offers insights for researchers and policy makers seeking to more accurately identify and measure elements of teen-targeted food marketing messages.

## 2. Methods

This survey comprised part of a larger study in which teenagers used a specially designed mobile app to collect all examples of teen-targeted food and beverage marketing that they encountered for 7 days. Methods for the broader study have been detailed elsewhere [10,11,14]. The survey design included clear and simple questions in appropriate language for 13–17-year-olds, and a variety of question types to engage the audience (i.e., multiple choice, forced ranking, and open and closed-ended questions). The survey was pilot-tested with a smaller group of teens and revised for clarity based on their feedback [14]. The self-reported nature of the data allowed teens to tell us about their media use and perceptions of food marketing content in a way that reflects their own lived experience [13]. Biases were minimized with straightforward and clearly worded questions. The survey was administered (online, via email link) to participating teens at the end of the 7-day data collection period, and collected demographic information, as well as inquiring about media use, opinions on teen-targeted food brands and products and advertising persuasiveness. Participants were screened for eligibility and then responses were anonymized; to ensure privacy, they could not be linked with data from the broader study. Ethics was granted by the Conjoint Faculties Research Ethics Board at the University of Calgary (REB19-0020).

Data were collected between October 2022 and May 2024. Participants were recruited through Instagram (Canada-wide), as well as through schools (via teachers/classroom participation) and researcher networks in Alberta and Nova Scotia. Overall, 464 teens completed the survey. Some specific questions had a smaller sample size due to exclusions or missing answers. For example, “identify your most used media platform” had one answer excluded for an invalid response, while a question related to both teen-targeted food or beverage brands and time spent on media platforms had several missing answers (total sample sizes are noted in each table in the Results (Section 3)).

### 2.1. Survey Measures

Sociodemographic measures in the study included age (13–17), and gender (girl, boy, gender non-conforming). Outcome measures explored three areas: perceptions of teen-targeted food brands, media use, and persuasive power.

Teen perceptions of targeted food and beverage marketing were captured with the following (sequential) open-ended questions: “Are there any food or beverage brands (including fast food) that you think specifically target teenagers? (List the top three brands)” and “What makes the brands teen-targeted?”.

Media platform use and daily time spent were explored by asking teens to “*rank the top 3 media platforms that you use most often, using (1, 2, 3) to show the order of importance to you*”. Response options (entered as forced ranking for top three) included: *Instagram, Snapchat, TikTok, Twitter, Twitch, Facebook, YouTube, Website, Video Game, Outdoor Billboard, Sign/Poster, Sponsored Event/Team, TV, Magazines, Food or Beverage Packaging, or Other*. A follow-up question inquired: “*how many minutes a day do you spend on each of the top 3 platforms that you identified above?*” Response options were selected from a set list (developed and refined from pilot data) which included 1–30 min, 30–60 min, 1–2 h, 2–3 h, 3–4 h, 4–5 h, and more than 5 h.

Perceptions of persuasive power were explored by asking teens to identify the most important media platform (i.e., communication channel) when it comes to food marketing targeted at teenagers, as well as the most important persuasive marketing technique when it comes to identifying *what is*, or *what counts as*, a teen-targeted food advertisement. In both cases, a pre-set list of responses was provided of 16 platform options and 11 persuasive techniques/indicators (celebrity, cartoon character, humour, interactivity, language, music, special offer, teenaged actor, theme, visual style, and others). The list of platforms and persuasive techniques was developed and tested out of previous research [10,14].

### 2.2. Statistical Analysis

Survey responses, including sample characteristics and questions regarding media platforms, use and marketing techniques, were summarized using frequencies and percentages. Frequencies and any associations across genders and age groups (younger teens, 13–14 years; older teens, 15–17 years) were compared using chi-square tests for 3 questions (teen-targeted brands, most important platform, most important technique).

## 3. Results

Four hundred and sixty-four teenagers participated in this study. Most participants (71%) were older teenagers (ages 16–17) and girls (75%). Table 1 provides a breakdown of gender by age.

### 3.1. Most Often Used Media Platforms

Overall, Instagram was identified as the most popular platform by teenagers. It ranked first as both the number 1 and 2 most frequently used platform (44.6% and 29.1%). Table 2 provides the respondents’ ranked answers for the top three most used media platforms.

### 3.2. Time Spent on Media Platforms

**Figure 1:**
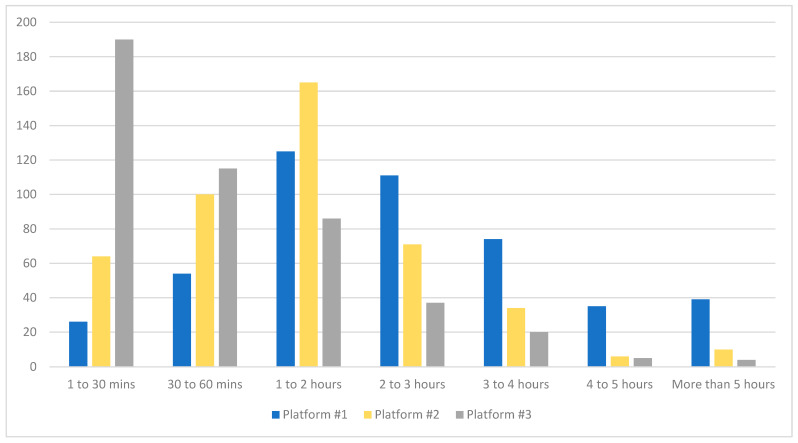
Daily time spent on the #1, #2, and #3 most used media platform, according to teens.

The most frequently identified time frame for daily use of the top two media platforms was 1 to 2 h (26.9%, 35.9%), while the #3 platform was most often used for 30 to 60 min per day (41.6%). Figure 1 compares daily use time frames between the #1, #2, and #3 most used platforms.

### 3.3. Food and Beverage Brands Targeting Teens

When asked to identify food or beverage brands targeting teenagers, teens responded with 199 unique responses. McDonald’s was the most frequently identified brand across all three categories (54.6%). Notably, the top four brands were consistently identified in the same ranking: (1) McDonald’s, (2) Starbucks, (3) Coca-Cola, and (4) Tim Hortons (a Canadian-based brand originating with coffee and donuts). Table 3 summarizes the top ten food or beverage brands identified by teenagers.

A significant association exists between the selection of first preference of the top four ranking brands and age group (*p* = 0.0191). Moreover, younger teens (ages 13–14) were statistically more likely to name Coca-Cola than older teenagers (ages 15–17), while older teens were more likely to identify Starbucks than younger teens. No associations were found related to gender and the top four identified food brands (*p* = 0.8807).

### 3.4. Reasons for Identifying Top Food or Beverage Brands as Teen-Targeted

When asked to explain why their selected food brands were “teen-targeted”, teenagers most frequently identified marketing techniques (i.e., ad content)—although the specific techniques varied depending on the brand (see Table 4, Table 5, Table 6 and Table 7). For example, special offer was most commonly associated with McDonald’s (22%), which participants explained as having “good deals on the app and free fry deals” (Girl, 17) or the “creation of celebrity-named meals” (G16), while Starbucks was most noted for visual style (37%), especially in association with “colourful and ‘trendy’ marketing” (G17), and “social media posts that use layouts that attract teens” (G17). Visual style was also a prominent technique named in relation to Coca-Cola (29%), as explained by their “animation and interactive effects” (G16) and “fonts that appeal to teens” (G17), while Tim Hortons was associated with celebrity (41%)—ones considered to attract teens so that “surely many teens would want to try that product” (G15).

Table 4, Table 5, Table 6 and Table 7 also reveal intriguing nuances pertaining to teenager perceptions and branding. Teenagers were asked to identify food and beverage brands that they thought “specifically targeted teenagers”. The term “targeted” would lead one to think of marketing strategies. Yet the reasons these food brands were “teen-targeted” extended beyond the persuasive appeals of advertising. They also had to do with pricing, the platform hosting the brand ad, the taste of the food and convenience. Such reasons equally varied by brand. Low price was deemed (in nearly one of every five answers) as the reason that McDonald’s is a teen-targeted food brand (“value-conscious meal options”, G15); a reason also identified in nearly one of every four answers for Tim Hortons (“cheap and affordable”, G17). Low price was not noted as a reason Starbucks or Coca-Cola was teen-targeted, however. Teens also identified convenience as a reason McDonald’s and Tim Horton’s were teen-targeted food brands (“quick and easy access” G15; “convenient locations around the city” G16), in proportions far exceeding those for both Starbucks and Coca-Cola. The taste of the food itself as a reason the brand was teen-targeted ranked highest for Coca-Cola (a “sweet drink”, G15; “lots of teens enjoy drinking [it]”, G16), whereas the social-emotional value of the brand was most commonly noted for Starbucks (“Popular with teens… [and the] general culture around the brand [is teen-targeted]”, G16).

### 3.5. Media Platforms of Importance for Teen-Targeted Food Marketing

**Figure 2:**
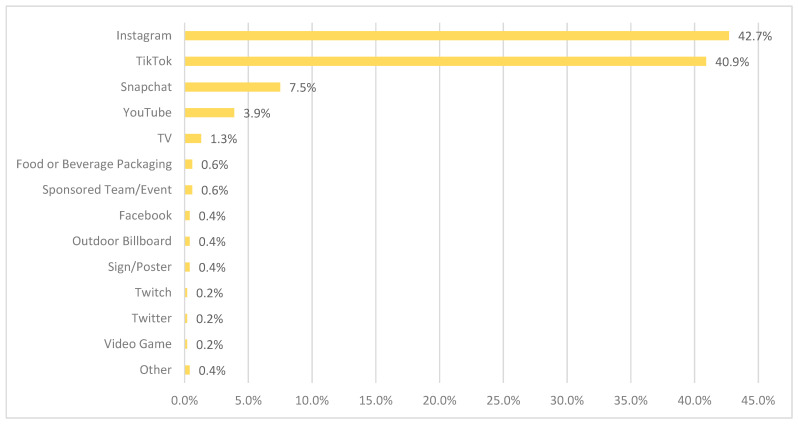
Most important media platform when it comes to teen-targeted food and beverage marketing, according to teens (*n* = 464).

Survey participants identified Instagram (42.7%) as the media platform of highest importance when it comes to teen-targeted food marketing, followed closely by TikTok (40.9%). These two platforms far outpaced all others when it comes to teen-identified platforms for food marketing—with television, packaging, sponsored team/event, Facebook, Twitch (and others) comprising 1 percent—or under—of answers. (See Figure 2 for a ranked list of media platforms.) Note that a chi-square test did not find any associations between the top two platform selections and age (*p*-value = 0.11), or gender (*p*-value = 0.2654).

### 3.6. Teen Appealing Marketing Techniques

**Figure 3:**
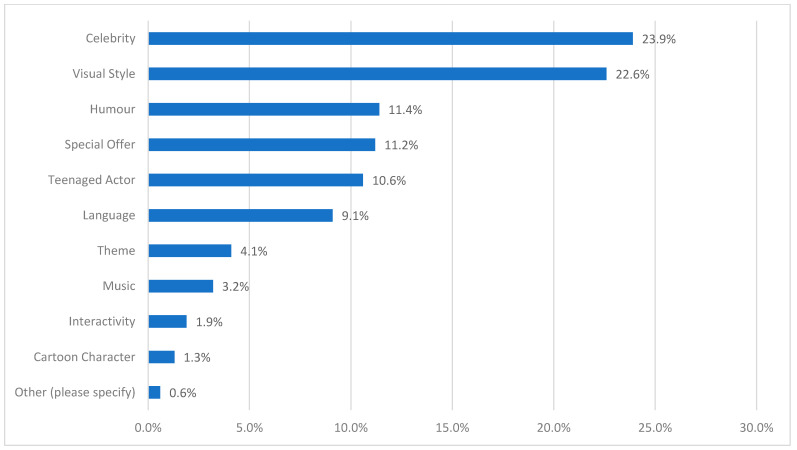
Most important marketing technique when it comes to teen-targeted food marketing, as identified by teens (*n* = 464).

Survey participants identified “celebrity” as the most important persuasive technique when it comes to teen-targeted food marketing (23.9%), followed closely by visual style (22.6%). Figure 3 provides ranked results for the complete list of marketing techniques (although no associations were found between the selection of the top four techniques and age (*p* = 0.6887), or gender (*p* = 0.965).

## 4. Discussion

This study provides a unique snapshot into teen perceptions of targeted food marketing, revealing that the platforms Instagram and TikTok, and specific persuasive techniques of celebrity and visual style are the most important to them. Persuasive marketing techniques comprise the “power” of a commercial message—they are the elements within an advertisement that make it compelling, according to the World Health Organization [15,16]. The concern with exploring persuasive power is the potential for creative content (i.e., the marketing techniques) to influence teen food attitudes and behaviours [17]. As outlined in the “hierarchy of unhealthy food promotion effects” model, this can take place through a cumulative “cascade of effects” that begins with brand awareness and positive attitudes toward the products [1] (p. e94). While investigation of specific elements of persuasive power is receiving more sustained treatment [10,11,18], the literature on food marketing more commonly focuses on advertising exposure [19,20,21,22,23,24]. Both approaches are necessary to gain a better understanding of the reach and power of food marketing directed at teens. Yet this survey adds to the understanding of power by asking teenagers important questions pertaining to salience—that is, it asks what elements of persuasive power are the most persuasive to them [11,25]. Here, we are employing the concept of salience as it relates to Robert Entman’s theory of framing from the field of communication, which (while primarily concerned with news coverage) underlines the importance of prominence when analyzing textual elements [11,25,26]. This matters because an advertisement may contain several persuasive appeals, but not all appeals will be equally persuasive. Probing what is salient to teenagers when it comes to marketing communication allows one to better understand, not only the types of appeals that resonate, but also what appeals they are more open (and vulnerable) to.

When it comes to assessing where teens see food marketing, survey studies include a range of settings or locations, from retail and school to online. Other survey studies have found the top location to be *movies and TV* (44%) for Canadian youth [12], or *grocery or convenience stores* (66%) for Australian youth [27]. However, our study is unique in that we focused on media platforms specifically, as opposed to settings or locations (such as stores), in order to assess what teens perceive as the most *important communication channel* related to teen-targeted marketing. Its findings emphasize the importance of digital platforms for teens: the top 4 answers were Instagram (43%), TikTok (41%), Snapchat (8%) and YouTube (4%), and provide insights into the specific media platforms (or apps) that teens see as directly relevant to teen-targeted food marketing. This provides a better understanding of how teens experience instances of food marketing (given the different platform/app designs) rather than providing catch-all categories such as “social media” or “online sources”. The particular importance of Instagram and TikTok as key sites of teen-targeted food marketing is also supported by other research [10,11,14].

While few studies on teenagers and food marketing explore its content, teen-specific marketing techniques are useful to gain a better understanding of how brands appeal to this market. Selecting from a list of teen-validated indicators from a related study [14], teens participating in our survey identified *celebrity*, *visual style* and *humour* as the top three most important marketing techniques related to teen-targeted food messaging. Other surveys have similarly identified the category of “famous people” in the top three marketing techniques selected by teens [12]. However, more research is needed to explore which specific celebrities appeal to teens, as previous food marketing research has focused on athlete endorsements [28,29], and does not take into account the range of singers/rappers, actors and influencers featured in food marketing that appeal to teens [10].

Understanding teen perceptions of food brands is another important facet in assessing the current landscape of teen-targeted food messaging. While other survey research has explored Canadian teen’s preference for specific popular food brands (McDonald’s, Subway, and KFC; with about three-quarters of teens indicating preference for the first two) [13], our study took a more direct approach to assessing brand perceptions by asking teens to identify their top 3 choices for teen-targeted food or beverage brands, and the reasons why. Our findings show a significant level of participant agreement when it comes to brands that are teen-targeted: the same four brands, McDonald’s, Starbucks, Coca-Cola, and Tim Hortons, were identified across three categories in the same ranked order. These findings provide important insight in terms of teen appeal since three of the four top identified brands are beverage-focused (Starbucks, Coca-Cola, Tim Hortons). The brands teens themselves identified in this study differ from the food categories being documented by adult researchers. For example, a previous Canadian study of websites popular with teenagers found that the food categories of cakes, cookies, and ice cream were the most commonly advertised [30]. Further, our study found an association between brand identification and age with younger teens being more likely to identify Coca-Cola as teen-targeted, with older teens more likely to identify Starbucks as teen-targeted, suggesting that age can be a relevant factor in the consideration of brand/food preferences. More research is needed to explore why older and younger teens feel this way. This study did not find any association with gender; elsewhere, however, such relationships between teens, gender and food marketing content are beginning to be explored [11,20,31,32].

All teens in our study indicated that ad content (i.e., presence of persuasive techniques) was the top reason for thinking a food/beverage brand was targeting them. Specifically, McDonald’s was most closely associated with *special offers* (i.e., discounts, limited-time offers), Starbucks and Coca-Cola with *visual style* (i.e., colours, fonts, design), and Tim Hortons with *celebrity* (i.e., actors, singers, athletes, influencers). These were the marketing techniques that teens identified as being the most salient to them, as represented in the content belonging to these specific brands. Yet, when identifying what makes such brands teen-targeted, teens also consider factors beyond marketing advertisement [33], leading to a more nuanced understanding of brand preferences. For example, teenagers pointed to the social importance of Starbucks for them, noting the brand’s popularity among their age group, as well as their interest in sugary, colourful, and trendy beverages (with “fun, fruity flavours”) such as refreshers and Frappuccinos. Similarly, teens’ clear preference for the visual style associated with the Coca-Cola brand in our study does not single-handedly account for why they thought the brand targeted teenagers, since the drink’s taste profile (i.e., sugary, caffeinated, carbonated) was also commonly noted.

Considering teen perceptions of food and beverage brands more broadly offers unique insights into teen appeal and the persuasive power of targeted food messaging, and raises additional questions around teen attitudes and behaviours. More specifically, further areas of research around questions of impacts include the relationship between teen perceptions of these brands and exposure (do they follow these brands, as well as see ads?); between these “teen appealing brands” and food preference (do they like them more because they appear to cater to teens?); and between these “teen appealing brands” and consumption (are they more likely to consume them because they perceive them as made for teens?). For policy makers, the question of salience around persuasive power is an important one, which can enrich monitoring efforts of specific targeted marketing techniques as identified by teens themselves. Further, a better understanding of teen views on the most persuasive elements of targeted marketing messages is very useful for educators invested in promoting media literacy and food marketing skills for this group to help them navigate a challenging food landscape.

## 5. Strengths and Limitations

Study strengths include the focus on Canadian teenagers and their perceptions of persuasive power around targeted food and beverage marketing. As exploratory research (with recruitment via an open-call ad online {Instagram, website} and snowball sampling) the study does not purport to be a nationally representative sample of teenagers across Canada, nor does it account for regional differences. This could be a productive avenue for future research. Note that teens self-reported media platform use in this study; as such, estimates may be higher or lower than the teenagers reported. This said, the self-reported measures are appropriate to the exploratory nature of the research; they reflect the lived experience of participants and allow for easier data collection [13], and they speak to the concept of salience that we explore in this study. Finally, our study describes differences in perceptions of food marketing among our sample, which were taken at face value as reported by participants. However, some differences may be due to underlying factors as we did not adjust for potential confounding variables.

## 6. Conclusions

This study contributes to the limited survey literature on teen food marketing, providing a unique focus on teen perceptions of food marketing platforms, content and “teen brands”. It underscores the importance of considering teen perceptions of the food messaging that targets them, revealing the relative importance of a range of marketing techniques, platforms and food/beverage brands to this age group. The results provide insights into how to more accurately identify and measure what counts as targeted food marketing to Canadian teenagers.

## Figures and Tables

**Table 1:** Participants by age and gender (*n* = 464).

Age	Gender	Number	Proportion%	Proportion%
13 years	Girl	4	0.9%	1.3%
	Boy	2	0.4%	
	Gender Non-Conforming	0	0.0%	
14 years	Girl	26	5.6%	7.8%
	Boy	8	1.7%	
	Gender Non-Conforming	2	0.4%	
15 years	Girl	70	15.1%	19.8%
	Boy	18	3.9%	
	Gender Non-Conforming	4	0.9%	
16 years	Girl	119	25.6%	34.3%
	Boy	25	5.4%	
	Gender Non-Conforming	15	3.2%	
17 years	Girl	127	27.4%	36.9%
	Boy	28	6.0%	
	Gender Non-Conforming	16	3.4%	

**Table 2:** Top 3 most used media platforms according to teens (*n* = 464).

	#1 Most Used Platform			#2 Most Used Platform			#3 Most Used Platform		
Rank	Platform	Number	Proportion%	Platform	Number	Proportion%	Platform	Number	Proportion%
1	Instagram	207	44.6%	Instagram	135	29.1%	YouTube	106	22.8%
2	TikTok	140	30.2%	TikTok	116	25%	Instagram	101	21.8%
3	Snapchat	57	12.3%	YouTube	81	17.5%	Snapchat	93	20%
4	YouTube	38	8.2%	Snapchat	76	16.4%	TikTok	45	9.7%
5	TV	4	0.9%	Twitter	11	2.4%	Website	21	4.5%
6	Website	4	0.9%	Video Game	10	2.2%	TV	19	4.1%
7	Video Game	3	0.6%	TV	9	1.9%	Twitter	18	3.9%
8	Food or Beverage Packaging	2	0.4%	Website	9	1.9%	Facebook	11	2.4%
9	Sign/Poster	2	0.4%	Facebook	5	1.1%	Food or Beverage Packaging	11	2.4%
10	Facebook	1	0.2%	Food or Beverage Packaging	4	0.9%	Sign/Poster	11	2.4%
11	Twitch	1	0.2%	Sign/Poster	2	0.4%	Video Game	11	2.4%
12	Twitter	1	0.2%	Twitch	1	0.2%	Outdoor Billboard	6	1.3%
13	Magazine	0	0	Magazine	0	0	Twitch	3	0.6%
14	Outdoor Billboard	0	0	Outdoor Billboard	0	0	Sponsored Event	1	0.2%
15	Sponsored Event	0	0	Sponsored Event	0	0	Magazine	0	0
16	Other	3	0.6%	Other	4	0.9%	Other	9	1.9%

**Table 3:** Top 10 food and beverage brands as identified by teens as the #1, #2 and #3 teen-targeted brand.

	#1 Food or Beverage Brand (*n* = 459)			#2 Food or Beverage Brand (*n* = 415)			#3 Food or Beverage Brand (*n* = 366)		
Rank	Brand	Number	Proportion%	Brand	Number	Proportion%	Brand	Number	Proportion%
1	McDonald’s	131	28.5%	McDonald’s	65	15.7%	McDonald’s	38	10.4%
2	Starbucks	73	15.9%	Starbucks	53	12.8%	Starbucks	29	7.9%
3	Coca-Cola	40	8.7%	Coca-Cola	29	7.0%	Coca-Cola	29	7.9%
4	Tim Hortons	19	4.1%	Tim Hortons	23	5.5%	Tim Hortons	22	6.0%
5	Prime	13	2.8%	Prime	15	3.6%	Subway	20	5.5%
6	Monster Energy	9	2.0%	Subway	13	3.1%	Burger King	12	3.3%
7	Doritos	9	2.0%	Monster Energy	10	2.4%	Taco Bell	8	2.2%
8	Oreo	7	1.5%	Hershey	9	2.2%	Wendy’s	7	1.9%
9	Sprite	7	1.5%	Wendy’s	9	2.2%	Monster Energy	6	1.6%
10	Sour Patch Kids	6	1.3%	Chatime	8	1.9%	Dairy Queen	6	1.6%

**Table 4:** Reasons why teens identified McDonald’s as a teen-targeted food/beverage brand.

Reason (*n* = 277, More than One Reason Could Be Provided)	Number	Proportion%
Marketing Techniques/Content (top 3: special offer, 22%; visual style, 18%, celebrity, 15%, *n* = 162)	138	49.5%
Low Price	52	18.6%
Appeal of Food Itself (i.e., taste)	33	11.8%
Accessibility/Convenience	31	11.1%
Ad Platform/Location	19	6.8%
Social/Emotional Value	1	0.4%
Other	3	1.1%

**Table 5:** Reasons why teens identified Starbucks as a teen-targeted food/beverage brand.

Reason (*n* = 180, More than One Reason Could Be Provided)	Number	Proportion%
Marketing Techniques/Content (top 3: visual style, 37%; theme, 24%, interactivity, 10%, *n* = 117)	111	61.7%
Ad Platform	21	11.7%
Social/Emotional Value	20	11.1%
Appeal of Food Itself (i.e., taste)	19	10.6%
Accessibility/Convenience	3	1.7%
Other	3	1.7%

**Table 6:** Reasons why teens identified Coca-Cola as a teen-targeted food/beverage brand.

Reasons (*n* = 109, More than One Reason Could Be Provided)	Number	Proportion%
Marketing Techniques/Content (top 3: visual style, 29%; special offer, 18%; celebrity, 14%, *n* = 74)	66	60.6%
Appeal of Food Itself (i.e., taste)	21	19.3%
Ad Platform/Location	20	18.3%
Accessibility/Convenience	1	0.9%

**Table 7:** Reasons why teens identified Tim Hortons as a teen-targeted food/beverage brand.

Reasons (*n* = 71, More than One Reason Could Be Provided)	Number	Proportion%
Marketing Techniques/Content (top 3: celebrity, 41%, special offer, 25%, visual style, 13%, *n* = 32)	31	43.7%
Low Price	17	23.9%
Accessibility/Convenience	10	14.1%
Appeal of Food Itself (i.e., taste)	5	7.0%
Ad Platform/Location	4	5.6%
Social/Emotional Value	4	5.6%

## Data Availability

The data set is available from the authors upon request, and will be accompanied by a formal data sharing agreement outlining that the data be used for academic purposes only. It is not publicly available for privacy reasons.
